# Machine learning analysis of sex and menopausal differences in the gut microbiome in the HELIUS study

**DOI:** 10.1038/s41522-024-00628-z

**Published:** 2024-12-19

**Authors:** Esther M. C. Vriend, Henrike Galenkamp, Hilde Herrema, Max Nieuwdorp, Bert-Jan H. van den Born, Barbara J. H. Verhaar

**Affiliations:** 1 Amsterdam UMC, University of Amsterdam, Department of Internal Medicine, Section Vascular Medicine, Amsterdam Cardiovascular Sciences, Amsterdam, The Netherlands; 2 Amsterdam UMC, University of Amsterdam, Department of Public and Occupational Health, Amsterdam Public Health Research institute, Amsterdam, The Netherlands; 3 Department of Experimental Vascular Medicine, Amsterdam UMC, Amsterdam, The Netherlands

**Keywords:** Microbiome, Gastrointestinal system

## Abstract

Sex differences in the gut microbiome have been examined previously, but results are inconsistent, often due to small sample sizes. We investigated sex and menopausal differences in the gut microbiome in a large multi-ethnic population cohort study, including 5166 participants. Using machine learning models, we revealed modest associations between sex and menopausal status, and gut microbiota composition (AUC 0.61–0.63). After adjustments for age, cardiovascular risk factors, and diet, a part of the associations of the highest-ranked gut microbes with sex were attenuated, but most associations remained significant. In contrast, most associations with menopausal status were driven by age and lost significance after adjustment. Using pathway analyses on metagenomic data, we identified sex differences in vitamin B6 synthesis and stachyose degradation pathways. Since some of sex differences in gut microbiome composition and function could not be explained by covariates, we recommend sex stratification in future microbiome studies.

## Introduction

Despite a growing interest in the impact of the gut microbiome on health and disease, sex differences in the field of microbiota composition remain understudied. Much of our knowledge on sex and menopausal differences in microbiota composition originates from animal studies^1–4^. These studies demonstrate that microbial enzymes contribute to sex hormone metabolism, while sex hormones conversely affect gut microbiota composition^5–7^. Human studies support associations between microbial steroid-degrading enzymes and circulating levels of sex hormones^8–10^, however, reports on sex differences in gut microbiome composition are inconsistent^11–21^. These inconsistencies may be explained by different methodologies in often relatively small populations or insufficient adjustment for demographic, dietary and lifestyle differences that may influence microbial differences between men and women.

Although sex is often included as a covariate in gut microbiome analyses^22,23^, its precise impact often remains unclear because of a lack of sex stratification. In contrast, dietary intervention studies that do stratify by sex or menopausal status have shown differential effects on the gut microbiome^24,25^. To reduce variability, many clinical trials exclude premenopausal women^26^, potentially limiting generalisability to broader populations. Hence, a better understanding of sex and menopausal differences in gut microbiota composition and function is needed for designing and interpreting microbiome studies.

In this study, we investigated the effect of sex and menopause on gut microbiome composition and function using XGBoost machine learning models, to determine the overall impact of sex and menopause and identify relevant microbes and pathways. We hypothesised that differences in gut microbiota composition related to sex and menopause would be modest and largely driven by differences in covariates, while we expected microbial pathway differences to be more pronounced and less affected by confounders.

## Results

### Study population

From the multi-ethnic population-based HELIUS cohort, we included 5166 participants with available faecal samples and no antibiotic use within three months prior to sample collection (Table 1)^27^. The median age of the population was 51.0 years [interquartile range (IQR) 42.0, 58.0], with women being on average 2 years younger than men. The ethnic backgrounds differed between men and women: more women were of African Surinamese origin, while men were more often of Dutch and Moroccan origin. Mean body mass index (BMI) was slightly higher in women, while the prevalence of smoking, hypertension and diabetes was higher in men. The dietary patterns showed clear sex differences, with higher energy and macronutrient intake (carbohydrates, proteins, fat, fibre) in men than in women. The median difference in age between the pre- and postmenopausal groups was 13 years, and the use of antihypertensive, lipid-lowering, and glucose-lowering medications was higher in postmenopausal compared to premenopausal women (Supplementary Table 1). The median time since menopause onset in postmenopausal women was 9.0 years [IQR 4.0, 15.0]. Differences in energy intake and macronutrients according to menopausal status were considerably smaller compared to differences between women and men. For fibre, fatty acids, and saturated fats, no significant differences were found between the pre- and postmenopausal groups (Supplementary Fig. 1).

**Table 1:** Population characteristics

	*n*	Men *n* = 2473	Women *n* = 2693	*P* value
Age (years)	5166	52.0 [43.0, 59.0]	50.0 [42.0, 58.0]	<0.001
Ethnicity	5166			<0.001
Dutch		749 (30.3)	709 (26.3)	
South-Asian Surinamese		309 (12.5)	333 (12.4)	
African Surinamese		522 (21.1)	749 (27.8)	
Ghanaian		210 (8.5)	266 (9.9)	
Turkish		238 (9.6)	247 (9.2)	
Moroccan		364 (14.7)	320 (11.9)	
Other		81 (3.3)	69 (2.6)	
BMI (kg/m^2^)	5161	26.6 ± 4.2	27.9 ± 5.4	<0.001
Current smokers	5135	660 (26.9)	402 (15.0)	<0.001
SBP (mmHg)	5161	132.6 ± 17.3	127.2 ± 18.6	<0.001
DBP (mmHg)	5161	83.8 ± 10.2	78.5 ± 10.4	<0.001
Hypertension	5161	1118 (45.2)	1023 (37.0)	<0.001
Antihypertensive medication	5166	503 (20.3)	630 (23.2)	0.008
Total cholesterol (mmol/l)	5151	5.0 ± 1.0	5.1 ± 1.0	0.018
LDL (mmol/l)	5125	3.2 ± 0.9	3.0 ± 0.9	<0.001
Lipid-lowering medication	5165	380 (15.4)	280 (10.4)	<0.001
Glucose (mmol/l)	5151	5.8 ± 1.3	5.4 ± 1.2	<0.001
Diabetes mellitus	5156	332 (13.5)	268 (10.0)	<0.001
Glucose-lowering medication	5166	227 (9.2)	221 (8.2)	0.233
Sodium intake (g)	1054	2.3 [1.8, 3.1]	1.9 [1.5, 2.4]	<0.001
Alcohol intake (g)	1307	1.7 [0.0, 14.0]	0.2 [0.0, 4.5]	<0.001
Total calories intake (kcal)	1307	2397.8 [1898.4, 3028.9]	1907.0 [1520.2, 2348.0]	<0.001
Fibre intake (g)	1307	26.1 ± 11.6	23.1 ± 8.9	<0.001
Postmenopausal status	2693	-	1207 (44.8)	
Hormone replacement therapy	2693	-	33 (1.2)	
Hormonal contraception	2693	-	336 (12.3)	

Data are shown as mean ± standard deviation, median [interquartile range], or *n* (%).

*IQR* interquartile range, *SD* standard deviation, *BMI* body mass index, *SBP* systolic blood pressure, *DBP* diastolic blood pressure, *LDL* low-density cholesterol.

### Sex and menopausal differences in gut microbiota composition and diversity

**Fig. 1:**
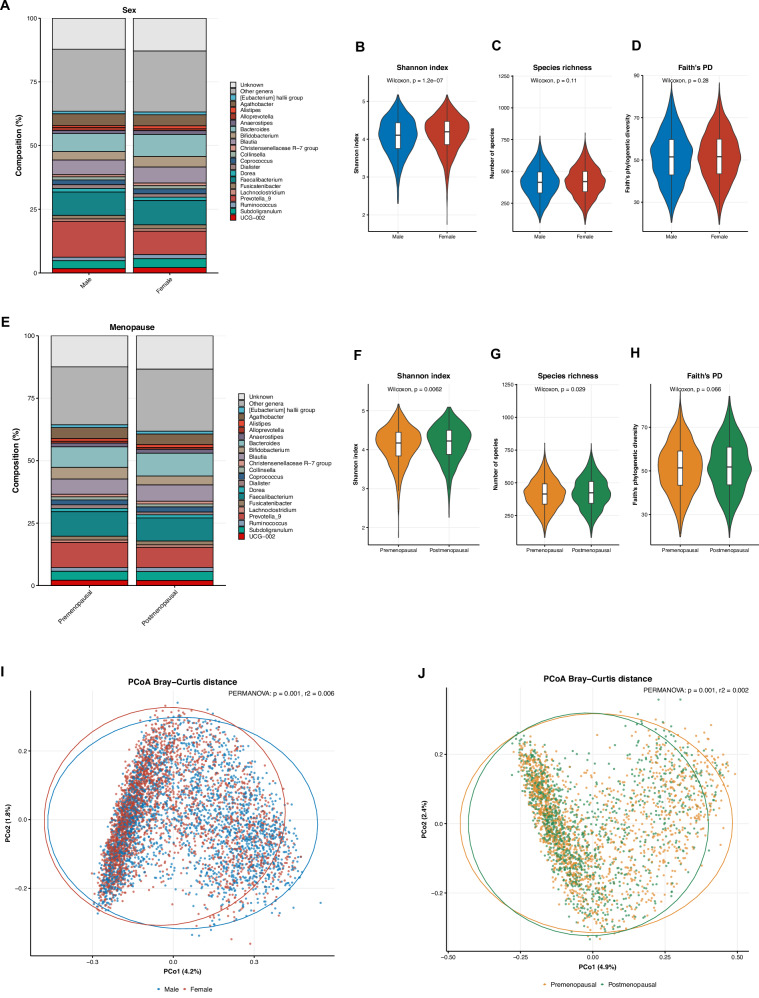
Descriptive differences in gut microbiota composition. **A** Gut microbiota composition at genus level for men and women; **B**–**D** Alpha diversity comparison between men and women; **E** Gut microbiota composition at genus level for premenopausal and postmenopausal women; **F**–**H** Alpha diversity comparisons between premenopausal and postmenopausal women; **I** Principal coordinate analysis (PCoA) of Bray–Curtis distance, comparison between men and women; **J** PCoA of Bray-Curtis distance, comparison between premenopausal and postmenopausal status. PD phylogenetic diversity, PCoA principal coordinates analyses, PERMANOVA permutational analysis of variance.

Faecal samples of the HELIUS participants were sequenced using 16S rRNA sequencing. We first assessed sex and menopausal differences in alpha and beta diversity. We found no sex differences in species richness and Faith’s phylogenetic diversity (*P* > 0.05; Fig. 1). However, the Shannon index for alpha diversity was significantly higher in men than in women, albeit with small absolute differences. Similarly, beta diversity as calculated with Bray-Curtis distance was significantly different between men and women (PERMANOVA: *P* = 0.001), but with an *R*^2^ of only 0.6%. In postmenopausal women, the Shannon index and species richness were marginally higher than in premenopausal women. Additionally, beta diversity differed significantly between pre- and postmenopausal women (PERMANOVA: *P* = 0.001), but with a low *R*^2^ of 0.2% (Fig. 1).

### Predicting sex from gut microbiota with machine learning models

**Fig. 2:**
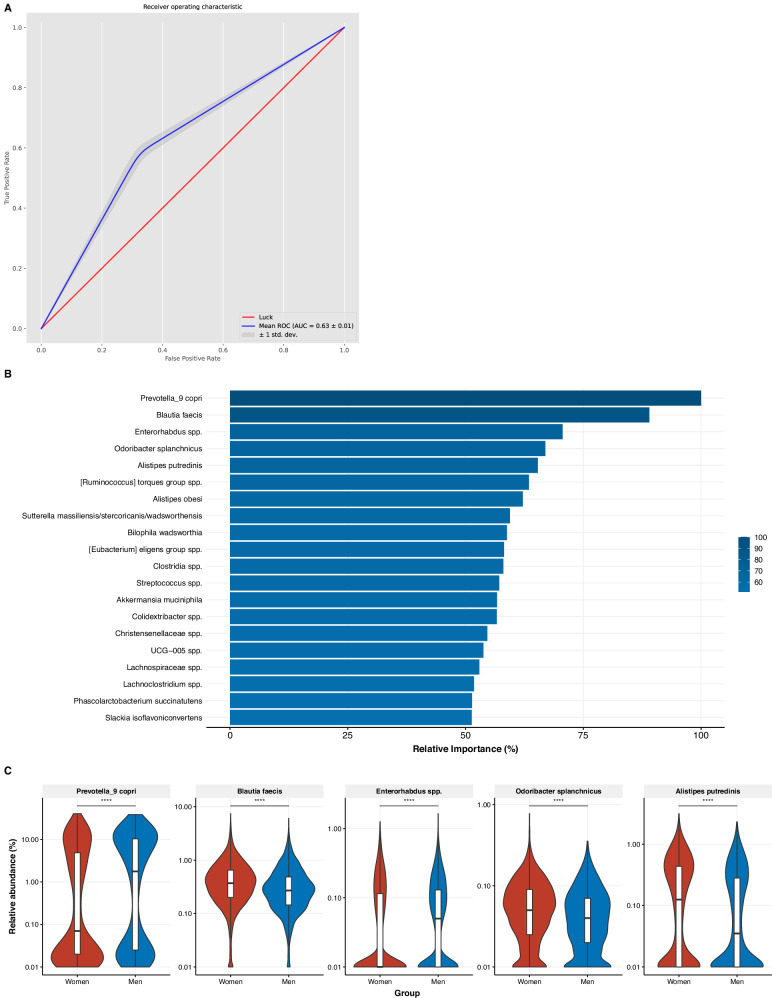
Machine learning model predicting sex from microbiota composition. **A** Area under the curve (AUC) of the classification model predicting sex from gut microbiota composition; **B** Relative feature importance resulting from the machine learning prediction (first feature always set at 100%, other features shown relative to first); **C** Sex differences in relative abundances (log10-scale) of highest ranked predictors tested with Mann-Whitney *U* tests.

To assess differences in gut microbiota composition between men and women, we used a machine learning model using the XGBoost algorithm in a nested cross-validation design to predict sex from gut microbiota composition. This model yielded an area under the curve (AUC) of 0.63 ± 0.01 (Fig. 2), indicating a modest association. The highest-ranked gut microbial predictors for sex in this model included *Prevotella_9 copri*, *Blautia faecis*, *Enterorhabdus* spp., *Odoribacter splanchinicus*, *Alistipes putredenis*, and *Ruminicoccus torques group* spp., with higher relative abundances in women, except for the *Prevotella_9 copri* and the *Enterorhabdus* spp. that were more abundant in men. Since the AUC could have been affected by combining premenopausal and postmenopausal women into one group, we also performed sensitivity analyses with men versus premenopausal women, and men versus postmenopausal women. However, both models yielded AUCs of 0.60 ± 0.01, illustrating that menopausal status did not obscure the sex differences in the first model.

For associations with the 20 highest-ranked predictors, we performed linear regression analyses to obtain effect sizes and to adjust for relevant covariates. The associations with sex remained largely unchanged after adjustments for age, BMI, hypertension, diabetes mellitus, and smoking (Fig. 4A). Further adjustment for diet attenuated some of the associations, leading to the loss of significance for 6 out of the 20 predictors. Additional adjustment for use of medication (antihypertensive, glucose-lowering, lipid-lowering medications, proton-pump inhibitors, and systemic steroids) as well as protein and saturated fat intake yielded similar findings and excluding participants using hormonal contraceptives yielded similar results (data not shown). No interactions between sex and ethnicity were found.

### Predicting menopausal status from gut microbiota with machine learning models

**Fig. 3:**
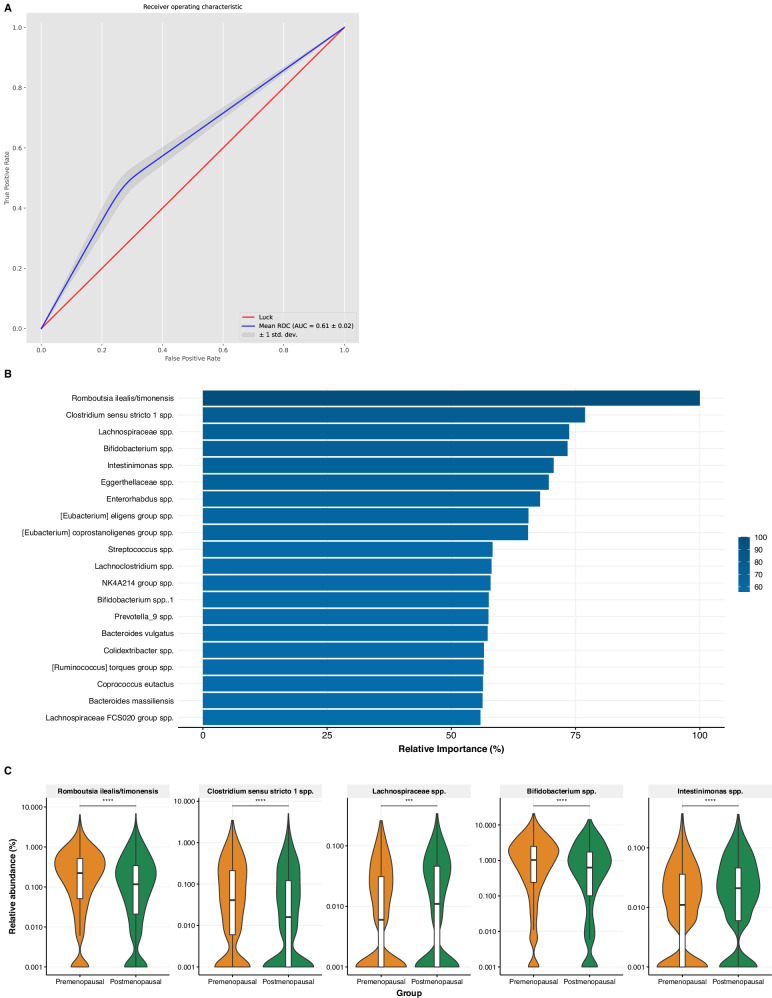
Machine learning model predicting menopausal status from microbiota composition. **A** Area under the curve (AUC) of the classification model predicting menopausal status from gut microbiota composition; **B** Relative feature importance resulting from the machine learning prediction (first feature always set at 100%, other features shown relative to first); **C** Menopausal differences in relative abundances (log10-scale) of highest ranked predictors.

**Fig. 4:**
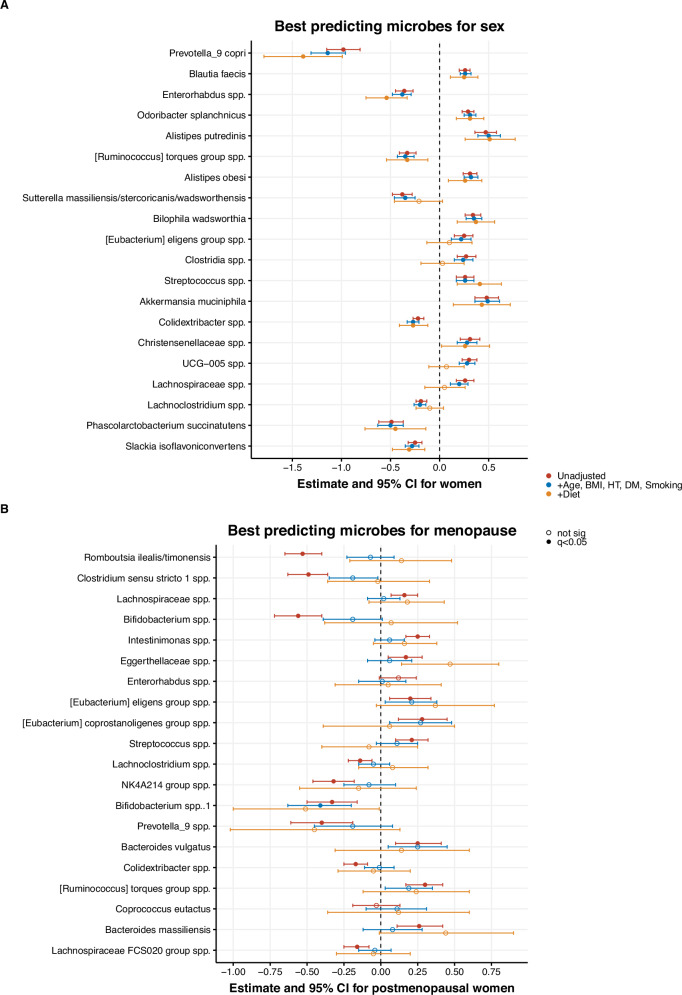
Linear regression models of best predicting microbes for sex and menopause. Forest plots of linear regression models with the sex or menopausal status as predictors and log-transformed microbe as outcome, showing (**A**) the best-predicting microbes for sex and (**B**) the best-predicting microbes for menopausal status, both ordered by feature importance in the machine learning model. Estimates with 95% confidence intervals as error bars. Model 1 is unadjusted. Model 2 is adjusted for age, BMI, hypertension (HT), diabetes mellitus (DM) and smoking. Model 3 is additionally adjusted for diet (total calories, fibre, proteins, sodium, and alcohol intake). *q*-values: *p*-values adjusted using the Benjamini-Hochberg procedure, *q* < 0.05 was considered significant.

We additionally investigated the associations between gut microbiota composition and menopausal status using a machine learning model with the same design, which resulted in an AUC of 0.61 ± 0.02 (Fig. 3). In the unadjusted linear regression models, differences in abundance of ASVs between the pre- and postmenopausal group were relatively small. Among the top 20 predictors for menopausal status were *Romboutsia ilealis/timonensis*, *Clostridium sensu stricto 1* spp., *Lachnospiraceae* spp., *Bifidobacterium* spp. and *Intestinimonas* spp., with higher relative abundances in premenopausal women, except for the *Lachnospiraceae* spp. and *Intestinimonas* spp. However, many of the initial significant associations disappeared after adjustment for relevant covariates, with the largest effects for age and diet (Fig. 4B). One significant interaction between menopausal status and ethnicity was observed between the African Surinamese and Dutch origin subgroups: *Enterorhabdus* spp. showed a weaker association with menopausal status for African Surinamese women compared to their Dutch counterparts (interaction term: *P* < 0.001).

### Sex and menopausal differences in functional pathways

Shotgun metagenomics data were available for a subset of 259 participants, allowing us to additionally investigate in the effect of sex and menopause on functional gut microbial pathways. Compared to all participants included in the 16S rRNA analyses, those with metagenomics data were slightly older and had a higher prevalence of diabetes, hypertension, and smoking (Supplementary Table 2). The models using compositional metagenomic profiles yielded comparable results as the 16S rRNA analyses for the associations with sex and menopausal status, albeit with wider confidence intervals due to smaller group sizes (Supplementary Figs. 2 and 3). The top predicting species for sex in the compositional analysis were *Akkermansia municiphila*, *Roseburia inulinivorans*, and *Prevotella copri clade A*, largely in line with the 16S analyses. *Akkermansia municiphila* was lower abundant in men compared to women, while *Roseburia inulinivorans* and *Prevotella copri clade A* showed higher relative abundances in men. In line with the 16S analyses, the associations between the top 20 predictors and sex were attenuated after adjustment for covariates (Supplementary Fig. 3).

**Fig. 5:**
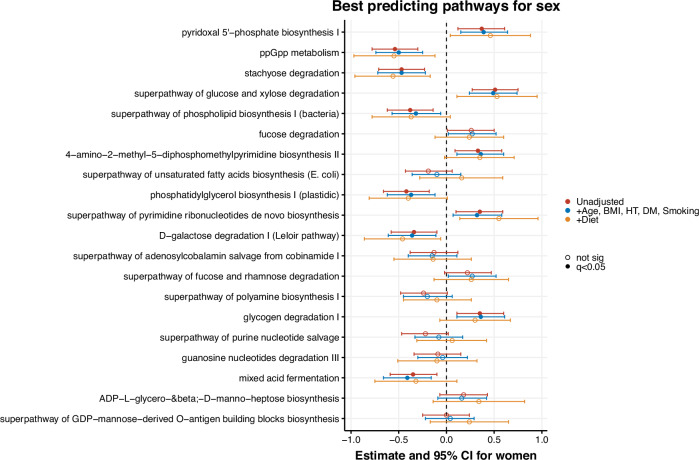
Linear regression models of best-predicting pathways for sex. Forest plots of linear regression models with the pathways (abundance scaled to zero-mean-unit-variance) as predictors and sex as outcome, showing the best predicting pathways for sex ordered by feature importance in the machine learning model. Estimates with 95% confidence intervals as error bars. Model 1 is unadjusted. Model 2 is adjusted for age, BMI, hypertension (HT), diabetes mellitus (DM) and smoking. Model 3 is additionally adjusted for diet (total calories, fibre, proteins, and alcohol intake). *q*-values: *p*-values adjusted using the Benjamini-Hochberg procedure, *q* < 0.05 was considered significant.

The model predicting sex from microbial pathways resulted in an AUC of 0.60 ± 0.06, comparable to the model using microbial species. The best-predicting features included pathways involved in pyridoxal 5’-phosphate biosynthesis I, guanosine tetraphosphate and pentaphosphate (ppGpp) metabolism, and the stachyose degradation pathway (Supplementary Fig. 4). Six of the top 20 predictors are pathways involved in the carbohydrate degradation. In the unadjusted linear regression models, 11 out of the top 20 predictors were significantly associated with sex. All of these associations persisted after further adjustment for age, BMI, hypertension, diabetes mellitus, and smoking (Fig. 5). The adjustment for dietary factors did not change the sex differences substantially, although the lower power of this model (*N* = 105) resulted in a loss of significance of the remaining associations. While the ppGpp and stachyose degradation pathways were lower in women, the pyridoxal synthesis (vitamin B6) was higher in women than in men. For pyridoxal synthesis, this sex difference appeared to be specifically driven by *Bacteroides ovatus*, *Bacteroides thetaiotaomicron*, and *Bacteroides uniformis* when assessing the stratified pathway data (per species; Supplementary Fig. 5). The stachyose degradation pathway was mostly explained by sex differences in this pathway in *Ruminococcus torques*, *Roseburia inulinivorans*, and *Roseburia intestinalis*. The sex difference in ppGpp metabolism could not be attributed to species with known taxonomy; unclassified species were driving this difference.

For menopausal status, both the machine learning models for composition and pathways did not result in associations that could be translated to a linear regression model (Supplementary Table 3, Supplementary Figs. 6–9). When stratifying pathways that showed sex differences for menopausal status, we found that the pyridoxal synthesis pathway was more abundant in postmenopausal compared with premenopausal women (Supplementary Fig. 10). However, the abundance of this pathway was also higher in men over 50 years compared with men younger than 50 years, likely reflecting an effect of age rather than hormonal status. The ppGpp metabolism and stachyose degradation pathways did not show differences between pre- and postmenopausal women, nor differences between men over and under 50 years of age.

## Discussion

We found modest differences in gut microbiota composition between men and women, and pre- and postmenopausal women. Our regression models showed that a part of the sex differences could be explained by diet, while differences between pre- and postmenopausal women could largely be attributed to age. Although microbial pathways showed sex differences that were less affected by covariates, the overall association between sex and microbial pathways was comparable in magnitude to that of microbial composition, indicating modest effects. Importantly, we found sex differences in vitamin B6 synthesis and stachyose degradation pathways, underscoring that even modest sex differences in gut microbiota composition could be biologically relevant when focusing on specific mechanisms.

Sex differences in gut microbiota composition have been documented in both animal and human studies, but often with smaller sample sizes, different methodologies, and varying attention to covariates^12–14,20^. Using a machine learning approach, we obtained one model metric that showed that the impact of sex on gut microbiota composition is relatively modest. In line with our results, the LifeLines-DEEP study including 1135 participants reported a higher microbial alpha diversity in women, and a higher abundance of *Akkermansia municiphila* in women compared to men^13^. The higher abundance of *Prevotella* spp. in men and increased abundance of *Ruminococcus* spp. in women, were also consistent with other studies^14,18^. In studies with smaller sample sizes no significant sex differences were found, or could only be attributed to covariates including differences in body composition^10,16^. These findings underscore the need for relatively high sample sizes to identify modest associations.

Many associations with menopausal status could be explained by age and lifestyle factors in our study, suggesting that environmental factors significantly influence menopausal differences in the gut microbiome. In addition, the overall association between gut microbiota composition and menopausal status was very modest in our machine learning model, consistent with the low explained variance of beta diversity by menopausal status. A previous study using metagenomic sequencing data of 2300 subjects, found that postmenopausal women are more similar to men in terms of alpha and beta diversity compared to premenopausal women^28^. In contrast to their findings, potentially due to different analysis approaches, we showed identical AUCs to discriminate men from premenopausal and postmenopausal women, underscoring the effect of sex.

Our analyses confirm that dietary patterns are an important contributor to differences in gut microbiota composition between men and women. Dietary factors, in particular fibre, animal protein and salt intake are known to shape the composition and function of the gut microbiome^11^. With our analyses, we have shown that a substantial part of the observed associations diminished after adjustment for dietary macronutrients, in line with the clear sex differences in dietary intake. For the associations with menopausal status, age had the largest impact. The relationship between age and microbiota composition is well established, as evidenced by previous studies examining microbiota variations along the lifespan^29^.

We used pathway analyses of metagenomic data to gain more mechanistic insight in the sex and menopausal differences and found the pyridoxal 5′-phosphate (vitamin B6) biosynthesis I pathway to me more abundant in women. Sex differences in this pathway are consistent with the reported menopausal differences in a smaller study including 89 women^21^. Both the human liver and bacteria require vitamin B6 for oestrogen degradation, contributing to higher circulating vitamin B6 levels in women compared to men. Additionally, vitamin B6 shows sex-specific effects on the microbiome, as for instance vitamin B6 supplementation increased *Akkermansia muciniphila* abundance in female but not male mice^30^. Similarly, in our data, the pyridoxal 5′-phosphate biosynthesis I pathway correlated with *Akkermansia muciniphila* abundance, and indeed only in women, illustrating that the vitamin B6 effects may also apply to humans. We also found sex differences in carbohydrate degradation pathways, including stachyose degradation. In mice, stachyose has been shown to stimulate the production of short chain fatty acids and the growth of *Akkermansia* and *Bifidobacterium*^31^. Its degradation process depends on the enzyme α-galactosidase from the glycoside hydrolase enzyme family. However, evidence regarding a potential interaction between sex hormones and α-galactosidase is still limited.

We could not confirm findings on sex and menopausal differences in steroid metabolism pathways in our cohort. These pathways and the 3-beta hydroxysteroid dehydrogenase enzyme are of relatively low prevalence and abundance, with previous studies showing small differences or only modest significance^8,9,21^. As such, our analysis approach using functional profiling might not have been sufficiently sensitive to detect those specific differences. In contrast, the pathway containing the beta-glucuronidase enzyme, which also plays a role in sex hormone metabolism^5^, was relatively abundant, but did not appear among the top predicting pathways for sex nor menopause.

Strengths of this study include the use of 16S sequencing data in a large, multi-ethnic, population-based cohort, while using metagenomic sequencing data for pathway analyses in a smaller subgroup, allowing for a deeper exploration of functional pathways. Our machine learning models enabled us to assess the overall impact of sex and menopause on the gut microbiota, while a cross-validation design, addition of random variables and a permuted set-up prevented overfitting. The robustness of our findings is supported by the consistency between results from 16S and metagenomic analyses. Because of the detailed phenotyping of these participants in the HELIUS cohort, we could assess the effect of a range of covariates on sex and menopausal differences, including medication use and dietary intake.

Limitations of our study include the absence of data on serum hormonal levels, since hormonal differences within same-sex individuals could have influenced the results. The classification of menopausal status relied solely on questionnaire responses, whereas direct measurement of hormonal levels would have provided more accurate categorisation. The AUCs resulting from the machine learning models were modest (0.60–0.63), which means that the overall impact of sex and menopause was limited, and downstream results should be interpreted with caution. In the regression models, even after adjusting for the most established covariates, there likely is residual confounding that might explain (a part of) the observed sex differences. While we tested whether the observed associations were attenuated after adjusting for dietary macronutrients, we did not include detailed dietary data at the level of specific food types. In addition, dietary data were collected in a subset of the HELIUS population, which resulted in smaller sample sizes in the diet-adjusted models.

Given the modest yet consistent gut microbiome differences between men and women in our cohort, we recommend sex stratification in future microbiome studies to be able to assess differential impacts of gut microbiota interventions. The weak associations between menopausal status and both gut microbiota composition and functional pathways indicate a limited impact of menopausal status, further supporting the inclusion of premenopausal women in clinical microbiome research. Longitudinal analyses could provide further insight on the changes in the gut microbiome during menopause, by measuring sex hormone (oestrogen, testosterone) levels. To improve understanding of the mechanisms behind sex differences in gut microbiota composition, in vitro studies could investigate whether sex hormones could directly modulate bacterial metabolism and growth, or indirectly, by shaping the host environment (including pH, mucus composition, immune responses).

Our analyses show that sex differences are more pronounced than menopausal differences in gut microbiome composition and functional pathways. Although the impact of sex on the gut microbiota was modest, a part of the sex differences could not be explained by differences in covariates. These findings are mechanistically incompletely understood, and underscore the importance of including sex stratification in future microbiome studies.

## Methods

### Study population

We used baseline data from the HEalthy Life in an Urban Setting (HELIUS) study, a prospective multi-ethnic population-based cohort study conducted in Amsterdam, the Netherlands. Detailed information regarding the HELIUS study has been described elsewhere^27^. Participants from six distinct ethnic backgrounds, aged between 18 and 70 years, were randomly sampled and invited from the municipality register, with stratification based on ethnic origin. We selected all participants with available baseline and gut microbiome data. Written informed consent was obtained from all participants, and the study received approval from the medical ethical review board of the Amsterdam UMC, location AMC. In this study, researchers adhered to the principles outlined in the Declaration of Helsinki. In writing this report, we followed the Strengthening the Reporting of Observational Studies in Epidemiology (STROBE) guidelines^32^.

### Measurements and definitions

Information on demographics, alcohol use and smoking status, medical history, and the use of medication was obtained through questionnaires. Fasting plasma samples were used to measure the concentrations of creatinine, total cholesterol, low-density lipoprotein (LDL) and high-density lipoprotein cholesterol (HDL), and glucose levels. A history of cardiovascular disease (CVD) was defined as a self-reported history of stroke, myocardial infarction, or coronary or peripheral revascularization. Blood pressure (BP) was measured twice on the left arm of the participants while seated, following a minimum of 5 min of rest, using a validated semi-automatic oscillometric device (Microlife WatchBP Home; Microlife AG, Switzerland). The average of two measurements was used to determine systolic and diastolic BP. Hypertension was defined as elevated BP levels (≥140/90 mmHg) or the use of BP-lowering medication. Diabetes mellitus was defined as elevated fasting glucose levels (≥7 mmol/l) and/or the use of glucose-lowering medication. Participants were asked to bring their prescribed medication to the research location, which was coded based on the Anatomical Therapeutic Chemical (ATC) classification system. Oral contraceptive and hormone replacement usage were determined based on self-reported medication use. Data on menopausal status was collected through questionnaires. Postmenopausal status was defined as the presence of 12 consecutive months of amenorrhoea with exceptions for pregnant or breastfeeding women and those using birth control. We used Food Frequency Questionnaires (FFQs) to record dietary intake, which have been validated in this multi-ethnic population^33^. From the FFQs, the intake of total calories, macronutrients (including sodium and fibre) and alcohol was calculated based on a nutrient database derived from the Dutch Composition Table 2011.

### Gut microbiota composition

Participants were asked to bring a morning stool sample within 6 h after collection. If this was not possible, they were advised to store the sample in a freezer overnight. The faecal samples were temporarily stored at −20 °C for a maximum of one day at the research site before being transferred to the central freezer (−80 °C) at the Amsterdam UMC (location AMC). Samples obtained from participants with diarrhoea in the week prior to collection or used antibiotics within three weeks prior to collection were excluded.

For 16S rRNA sequencing, the samples were shipped to the Wallenberg Laboratory at the Sahlgrenska Academy (University of Gothenburg, Sweden). DNA was extracted from a 150-mg aliquot using the repeated bead-beating method^34^. An Illumina MiSeq (Illumina RTA v.1.17.28; MCS v.2.5, San Diego, CA, USA) was used to determine the composition of the faecal microbiota by sequencing the V4 region of the 16S rRNA gene. Dual-indexing was achieved using 515F and 806R primers, together with the V2 Illumina kit for 2 × 250 bp paired-end reads^35^. PCR was conducted in duplicate reactions^36^. Sequencing reads were processed using a publicly shared Nextflow workflow (v.0.7) to infer amplicon sequence variants (ASVs; see ‘Code Availability)^37^. Using VSEARCH (v.2.27.0), fastqs were merged (*fastq_mergepairs*) and quality filtered (*fastq_filter*), reads were dereplicated (*fastx_uniques*), denoising was performed with alpha 2 and minimum size of 8 to infer ASVs (*cluster_unoise*), and singletons (*sortbysize*) and chimeras (*uchime_denovo3*) were removed. Finally, reads were mapped to the ASVs (*usearch_global*, *--id 0.97*)^38^. Taxonomy was assigned with the *assignTaxonomy* function of the DADA2 package using the SILVA database (v.138.1). The count table was rarefied to 15,000 counts per sample, resulting in the exclusion of 14 samples due to insufficient counts. The workflow resulted in a dataset comprising 5166 samples and 7659 taxa.

Metagenomic sequencing in a subset of 259 subjects was performed by Novogene (Cambridge, UK) on an Illumina HiSeq platform with 150 bp paired-end reads. For processing the raw sequencing reads, we used a Nextflow workflow (v.0.5.0-beta) that was made publicly available (see ‘Code Availability’)^37^. Within this workflow, reads were checked with fastqc (v.0.12.1) and quality filtered with fastp (v.0.23.4), which included adaptor detection and removal, sliding window quality trimming (window size 4 bp, threshold quality score 15) and removal of short reads of less than 70 bp. Human reads were removed with bowtie2 (v.2.5.2) and samtools (v.1.18), with GRCh38 as reference genome. The unmapped reads were subsampled to 20 million reads per sample using seqkit (v.2.7.0). We used MetaPhlAn (v.4.0.5) and the ChocoPhlAn database (vJun23_202307) to profile the reads for microbial composition, and HUMAnN (v.3.8) with the UniRef90 (v.201901b, EC filtered) and ChocoPhlAn (vJun23_202307) databases to infer functional pathways ^39,40^.

### Machine learning models

We used machine learning models with the XGBoost algorithm to predict gut microbiota composition from sex and menopausal status^41^. Machine learning models can include numerous features simultaneously, in contrast to separate univariate tests, and algorithms like XGBoost are relatively robust to the non-normal distributions inherent to microbiome data. XGBoost is a widely used algorithm employing extreme gradient boosting, recognised for its accurate and efficient predictions across diverse omics analyses, including those focusing on the microbiome^42^. These classification models had a nested cross-validation design. First, the ASV table was filtered for ASVs with more than five counts (corresponding to a relative abundance of 0.00033%) in 30 percent of subjects, to ensure that only non-spurious microbes present in a significant minority of individuals were included. The dataset was randomly split into a test (20% of participants) and a train set (80% of participants) in each iteration, whereafter a five-fold cross-validation was applied within the training set to tune the model hyperparameters. The tuned model was tested once on the test set. As a benchmark, two random variables were added to the predictor data in each iteration. Model performance was evaluated using the area under the receiver-operator curve (AUC) as main model metric. In addition, each of the models resulted in a ranked list of ASVs with their relative importance to the prediction. The same models were used for the metagenomics compositional and pathway analyses, replacing the ASV table with a metagenomics composition or pathway abundance table. The compositional table was filtered for species with an abundance higher than 0.1% in 25% of subjects, while the pathway table was filtered for pathways with an average abundance higher than 50 counts per million (cpm). The machine learning models were implemented using python (v.3.7.4), XGBoost (v.0.90), numpy (v.1.16.4), pandas (v.0.25.1), and scikit-learn (v.0.21.2) packages.

### Statistical analysis

Baseline characteristics were presented as mean (SD), median [IQR] or *n* (%), stratified by sex and menopausal status. Alpha diversity metrics (Shannon index, species richness, Faith’s phylogenetic diversity) and beta diversity metrics (Bray–Curtis distance) were calculated, and visualised stratified for sex and menopausal status. We used linear regression models to assess whether associations between the top 20 predictors from the machine learning models persisted after adjusting for covariates. In these models, sex or menopausal status were independent variables and the microbial counts (log-transformed after adding 1 pseudocount) or pathway abundances (in cpm) were dependent variables. Adjustments were made for 1) age and cardiovascular risk factors (BMI, hypertension, diabetes mellitus and current smoking status), and 2) diet (salt, alcohol, fibre and total calorie intake). Effect sizes and 95% confidence intervals (95% CI) were visualized in a forest plot. We additionally tested for interaction with ethnicity in the models adjusted for age and cardiovascular risk factors. The same linear regression models were used for metagenomics data (log-transformed relative abundances (%) for composition and untransformed pathway abundances (cpm)). *P* values were adjusted for multiple comparisons using the Benjamini-Hochberg procedure, resulting in q-values. All analyses were performed using RStudio (v.4.0.3) with ggplot2 (v.3.3.6), forcats (v.0.5.1), dplyr (v.1.0.5), tableone (v.0.13.0), phyloseq (v.1.48.0), vegan (v.2.6-6.1), and ape (v.5.8) packages. All code has been shared in a public Github repository (https://github.com/esthervriend/sex-differences-microbiome).

## Supplementary information

Supplementary Information

## Data Availability

The HELIUS data are owned by the Amsterdam University Medical Centres, location AMC, Amsterdam, The Netherlands. Any researcher can request the data by submitting a proposal to the HELIUS Executive Board as outlined at http://www.heliusstudy.nl/en/researchers/collaboration. The HELIUS Executive Board will check proposals for compatibility with the general objectives, ethical approvals and provided informed consent for the HELIUS study. The 16S sequencing data and the metagenomic sequencing data are available in the European Genome-Phenome Archive (https://ega-archive.org/studies/EGAS00001002969).

## Code availability

The code of the bioinformatic processing is shared at Zenodo (VSEARCH workflow: 10.5281/zenodo.10553479, metagenomics workflow: 10.5281/zenodo.10663326). All programming code used in the data analyses has been shared in a public Github repository (https://github.com/esthervriend/sex-differences-microbiome).
