# Supplementary material for: Machine learning analysis of sex and menopausal differences in the gut microbiome in the HELIUS study
Source: NPJ Biofilms Microbiomes. 2024 Dec 19;10:152. doi: 10.1038/s41522-024-00628-z (PMC11659428; doi:10.1038/s41522-024-00628-z)
Supplement: Supplementary file 1 — Supplementary Information [file 41522_2024_628_MOESM1_ESM.pdf]

Machine Learning Analysis of Sex and Menopausal Differences in Gut Microbiome in the HELIUS Study

Esther M.C. Vriend[1,2], Henrike Galenkamp[2], Hilde Herrema[3], Max Nieuwdorp[1], Bert-Jan H. van den Born[1,2], Barbara J.H. Verhaar[1,2*]

Author affiliations:

[1]Amsterdam UMC, University of Amsterdam, Department of Internal Medicine, Section Vascular Medicine, Amsterdam Cardiovascular Sciences, Amsterdam, The Netherlands

[2] Amsterdam UMC, University of Amsterdam, Department of Public and Occupational Health, Amsterdam Public Health Research institute, Amsterdam, The Netherlands

[3] Amsterdam UMC, Department of Experimental Vascular Medicine, Amsterdam, Netherlands.

* Correspondence: b.j.verhaar@amsterdamumc.nl

Supplementary Table 1: Population characteristics of pre- and postmenopausal women

	n	**Premenopausal** *n = 1,486*	**Postmenopausal** *n = 1,207*	**P-value**
Age (years)	2,693	44.0 [35.0, 50.0]	57.0 [53.0, 63.0]	<0.001
Ethnicity	2,693			<0.001
Dutch		334 (22.5)	375 (31.1)	
South-Asian Surinamese		180 (12.1)	153 (12.7)	
African Surinamese		348 (23.4)	401 (33.2)	
Ghanaian		164 (11.0)	102 (8.5)	
Turkish		180 (12.1)	67 (5.6)	
Moroccan		246 (16.6)	74 (6.1)	
Other		34 (2.3)	35 (2.9)	
BMI (kg/m^2)	2,690	27.4±5.4	28.5±5.5	<0.001
Current smokers	2,677	228 (15.4)	174 (14.5)	0.557
SBP (mmHg)	2.690	122.8±17.6	132.6±18.5	<0.001
DBP (mmHg)	2,690	77.2±10.4	80.1±10.1	<0.001
Hypertension	2,690	411 (27.7)	612 (50.8)	<0.001
Antihypertensive medication	2,693	224 (15.1)	406 (33.6)	0.013
Total cholesterol (mmol/l)	2.685	4.8±0.9	5.4±1.0	0.013
LDL (mmol/l)	2,680	2.9±0.8	3.3±1.0	<0.001
Lipid-lowering medication	2,693	87 (5.9)	193 (16.0)	<0.001
Glucose (mmol/l)	2,685	5.2±1.0	5.7±1.3	<0.001
Diabetes mellitus	2,688	94 (6.3)	174 (14.5)	<0.001
Glucose-lowering medication	2,693	76 (5.1)	145 (12.0)	0.190
Sodium intake (g)	553	2.0 [1.5, 2.5]	1.8 [1.4, 2.3]	0.010
Alcohol intake (g)	673	0.0 [0.0, 2.1]	0.4 [0.0, 7.8]	<0.001
Total calories intake (kcal)	673	1960.5 [1607.2, 2437.8]	1861.5 [1474.7, 2254.7]	0.009
Fiber intake (g)	673	23.6±9.3	22.6±8.4	0.141
Time since menopause onset (years)	1,207	-	9.0 [4.0, 15.0]	
Hormone replacement therapy	2,693	13 (0.9)	20 (1.7)	0.097
Hormonal contraception	2,693	132 (8.9)	20 (1.7)	<0.001

Data shown as mean±standard deviation, median [interquartile range], or n (%). IQR = interquartile range. SD = standard deviation. BMI = body mass index. SBP = systolic blood pressure. DBP = diastolic blood pressure. LDL = low-density cholesterol.

Supplementary Table 2: Population characteristics of subgroup with metagenomics data

	n	**Men**<br>*n = 115*	**Women**<br>*n = 144*	**P-value**
Age (years)	259	56.0 [48.0, 61.0]	52.0 [44.0, 59.0]	0.019
Ethnicity	259			0.001
Dutch		39 (33.9)	48 (33.3)	
South-Asian Surinamese		51 (44.3)	37 (25.7)	
African Surinamese		25 (21.7)	59 (41.0)	
BMI (kg/m^2)	258	26.6 (4.0)	27.4 (5.0)	0.155
Current smokers	258	36 (31.3)	23 (16.1)	0.006
Systolic blood pressure (mmHg)	259	135.2 (17.6)	129.9 (20.2)	0.028
Diastolic blood pressure (mmHg)	259	84.4 (10.9)	79.3 (11.0)	<0.001
Hypertension	259	66 (57.4)	61 (42.4)	0.023
Antihypertensive medication	259	35 (30.4)	37 (25.7)	0.480
Total cholesterol (mmol/l)	259	5.1 (1.1)	5.1 (1.0)	0.928
LDL (mmol/l)	259	3.3 (1.0)	3.1 (0.9)	0.164
Lipid-lowering medication	259	27 (23.5)	19 (13.2)	0.047
Glucose (mmol/l)	259	6.0 (1.3)	5.5 (1.0)	<0.001
Diabetes mellitus	259	24 (20.9)	17 (11.8)	0.070
Glucose-lowering medication	259	18 (15.7)	14 (9.7)	0.211
Sodium intake (g)	105	2.4 (1.0)	1.9 (0.8)	0.003
Alcohol intake (g)	129	9.9 (14.6)	3.9 (8.6)	0.004
Total calories intake (kcal)	129	2472.3 (902.9)	1845.8 (655.4)	<0.001
Fiber intake (g)	129	23.9 (8.6)	20.0 (6.5)	0.004

Data shown as mean±standard deviation, median [interquartile range], or n (%). IQR = interquartile range.

IQR = interquartile range. SD = standard deviation. BMI = body mass index. LDL = low-density cholesterol.

Supplementary Table 3: Population characteristics pre- and postmenopausal women with metagenomics data

	n	**Premenopausal** *n = 72*	**Postmenopausal** *n = 72*	**P-value**
Age (years)	144	45.0 [37.5, 51.0]	58.0 [54.0, 62.0]	<0.001
Ethnicity	144			0.175
Dutch		29 (40.3)	19 (26.4)	
South-Asian Surinamese		18 (25.0)	19 (26.4)	
Moroccan		25 (34.7)	34 (46.2)	
BMI (kg/m^2)	143	26.3 (5.1)	28.5 (4.7)	0.007
Current smokers	143	11 (15.5)	12 (16.7)	1.000
Systolic blood pressure (mmHg)	144	123.3 (18.2)	136.5 (20.1)	<0.001
Diastolic blood pressure (mmHg)	144	76.2 (10.6)	82.4 (10.5)	0.001
Hypertension	144	21 (29.2)	40 (55.6)	0.002
Antihypertensive medication	144	9 (12.5)	28 (38.9)	0.001
Total cholesterol (mmol/l)	144	4.9 (0.9)	5.2 (1.1)	0.087
LDL (mmol/l)	144	3.0 (0.7)	3.3 (1.0)	0.064
Lipid-lowering medication	144	2 (2.8)	17 (23.6)	0.001
Glucose (mmol/l)	144	5.3 (0.9)	5.6 (1.1)	0.038
Diabetes mellitus	144	5 (6.9)	12 (16.7)	0.121
Glucose-lowering medication	144	3 (4.2)	11 (15.3)	0.049
Sodium intake (g)	58	1.9 (0.7)	1.8 (0.8)	0.441
Alcohol intake (g)	68	2.7 (6.1)	4.9 (10.3)	0.302
Total calories intake (kcal)	68	1931.3 (583.7)	1769.8 (712.7)	0.314
Fiber intake (g)	68	21.1 (6.7)	19.0 (6.2)	0.175
Duration of menopause (years)	72	-	10.0 [4.8, 16.2]	-
Use of hormone replacement therapy	144	1 (1.4)	0 (0.0)	1.000
Use of hormonal contraception	144	19 (26.4)	1 (1.4)	0.039

Data shown as mean±standard deviation, median [interquartile range], or n (%). IQR = interquartile range.

IQR = interquartile range. SD = standard deviation. BMI = body mass index. LDL = low-density cholesterol.

Supplementary Table 4: STROBE checklist

	Item No	Recommendation	Page No
**Title and abstract**	1	(*a*) Indicate the study's design with a commonly used term in the title or the abstract	1
		(*b*) Provide in the abstract an informative and balanced summary of what was done and what was found	2
**Introduction**			
Background/rationale	2	Explain the scientific background and rationale for the investigation being reported	3
Objectives	3	State specific objectives, including any prespecified hypotheses	3
**Methods**			
Study design	4	Present key elements of study design early in the paper	14
Setting	5	Describe the setting, locations, and relevant dates, including periods of recruitment, exposure, follow-up, and data collection	14
Participants	6	(*a*) *Cohort study*—Give the eligibility criteria, and the sources and methods of selection of participants. Describe methods of follow-up<br>*Case-control study*—Give the eligibility criteria, and the sources and methods of case ascertainment and control selection. Give the rationale for the choice of cases and controls<br>*Cross-sectional study*—Give the eligibility criteria, and the sources and methods of selection of participants	14
		(*b*) *Cohort study*—For matched studies, give matching criteria and number of exposed and unexposed<br>*Case-control study*—For matched studies, give matching criteria and the number of controls per case	N/A
Variables	7	Clearly define all outcomes, exposures, predictors, potential confounders, and effect modifiers. Give diagnostic criteria, if applicable	14-15
Data sources/ measurement	8*	For each variable of interest, give sources of data and details of methods of assessment (measurement). Describe comparability of assessment methods if there is more than one group	14-15
Bias	9	Describe any efforts to address potential sources of bias	17-18
Study size	10	Explain how the study size was arrived at	14
Quantitative variables	11	Explain how quantitative variables were handled in the analyses. If applicable, describe which groupings were chosen and why	17-18
Statistical methods	12	(*a*) Describe all statistical methods, including those used to control for confounding	18
		(*b*) Describe any methods used to examine subgroups and interactions	18
		(*c*) Explain how missing data were addressed	18
		(*d*) *Cohort study*—If applicable, explain how loss to follow-up was addressed<br>*Case-control study*—If applicable, explain how matching of cases and controls was addressed<br>*Cross-sectional study*—If applicable, describe analytical methods taking account of sampling strategy	N/A
		(*e*) Describe any sensitivity analyses	18

Continued on next page

**Results**			
Participants	13*	(a) Report numbers of individuals at each stage of study—eg numbers potentially eligible, examined for eligibility, confirmed eligible, included in the study, completing follow-up, and analysed	4
		(b) Give reasons for non-participation at each stage	4
		(c) Consider use of a flow diagram	N/A
Descriptive data	14*	(a) Give characteristics of study participants (eg demographic, clinical, social) and information on exposures and potential confounders	4
		(b) Indicate number of participants with missing data for each variable of interest	Table1
		(c) *Cohort study*—Summarise follow-up time (eg, average and total amount)	N/A
Outcome data	15*	*Cohort study*—Report numbers of outcome events or summary measures over time	N/A
		Case-control study—Report numbers in each exposure category, or summary measures of exposure	N/A
		Cross-sectional study—Report numbers of outcome events or summary measures	Table1
Main results	16	(*a*) Give unadjusted estimates and, if applicable, confounder-adjusted estimates and their precision (eg, 95% confidence interval). Make clear which confounders were adjusted for and why they were included	5-6, figures
		(*b*) Report category boundaries when continuous variables were categorized	N/A
		(*c*) If relevant, consider translating estimates of relative risk into absolute risk for a meaningful time period	N/A
Other analyses	17	Report other analyses done—eg analyses of subgroups and interactions, and sensitivity analyses	6-8
**Discussion**			
Key results	18	Summarise key results with reference to study objectives	9
Limitations	19	Discuss limitations of the study, taking into account sources of potential bias or imprecision. Discuss both direction and magnitude of any potential bias	12
Interpretation	20	Give a cautious overall interpretation of results considering objectives, limitations, multiplicity of analyses, results from similar studies, and other relevant evidence	12-13
Generalisability	21	Discuss the generalisability (external validity) of the study results	11
**Other information**			
Funding	22	Give the source of funding and the role of the funders for the present study and, if applicable, for the original study on which the present article is based	19

Supplementary Figure 1: Dietary data principal component analysis

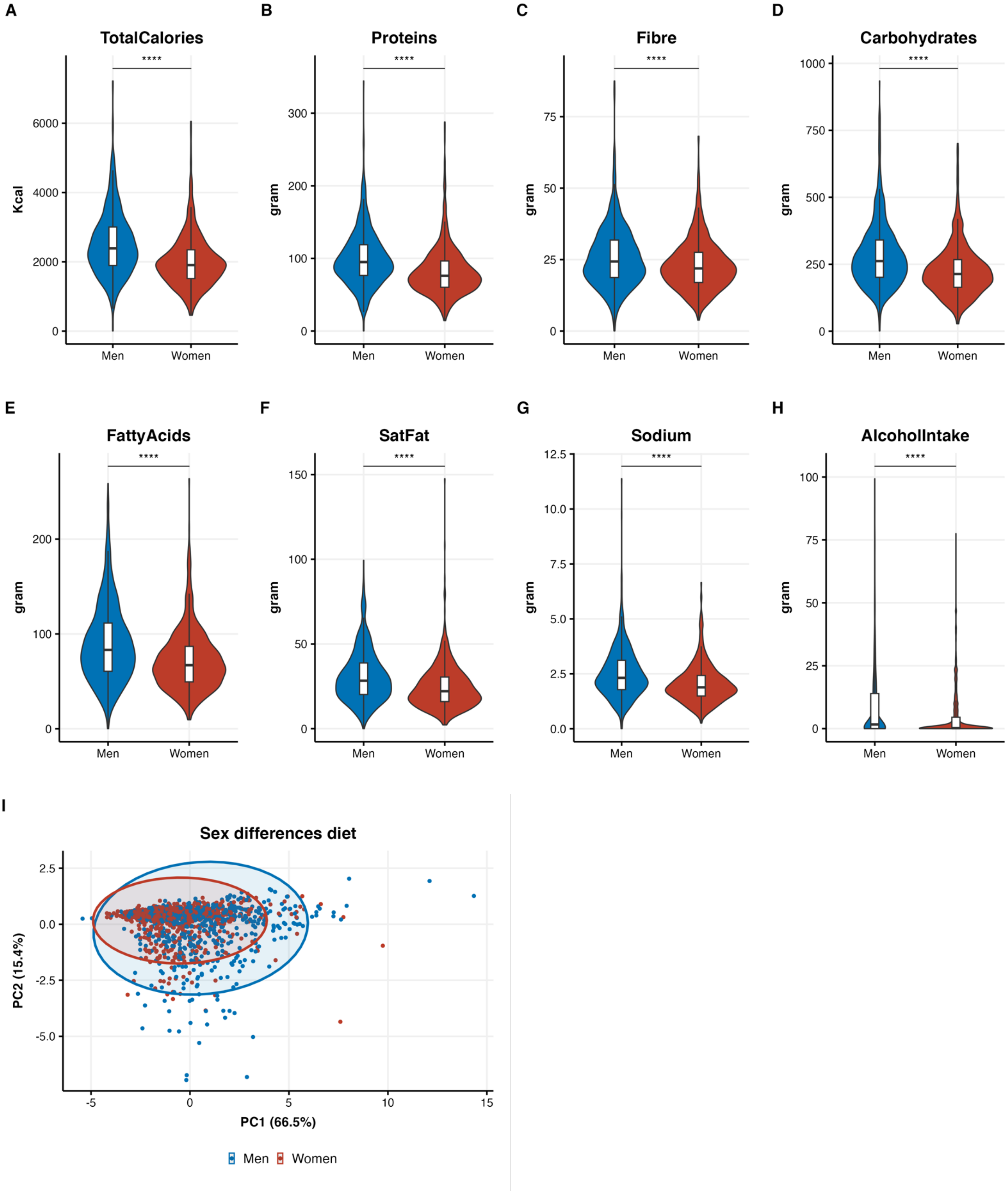

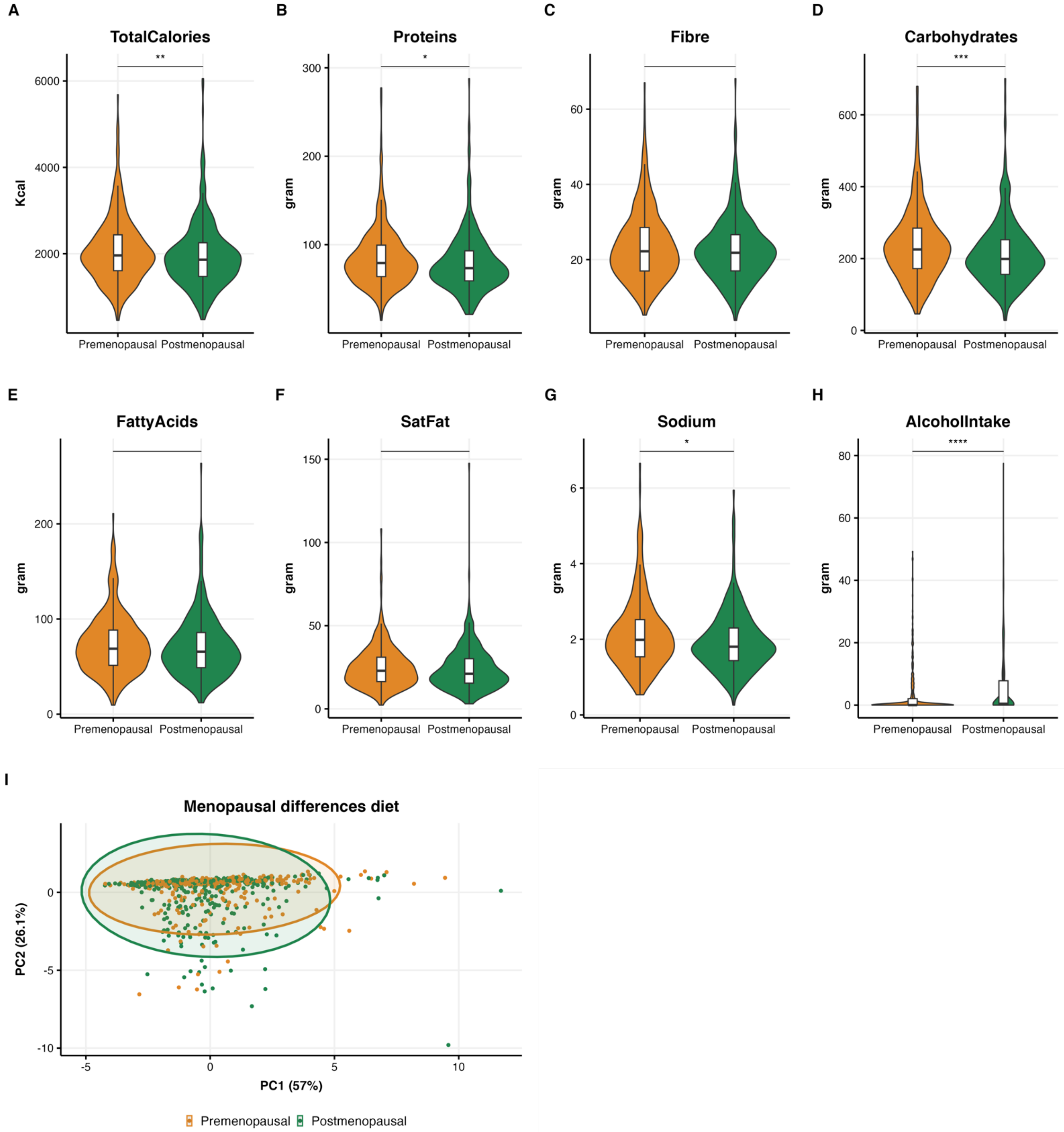

*Differences between men and women and pre- and postmenopausal women were tested with Mann Whitney U tests. * P-value < 0.05, ** P-value < 0.01,*** P-value < 0.001, **** P-value < 0.0001*

Supplementary Figure 2: Machine learning model: gut microbiota composition (metagenomic sequencing) and sex

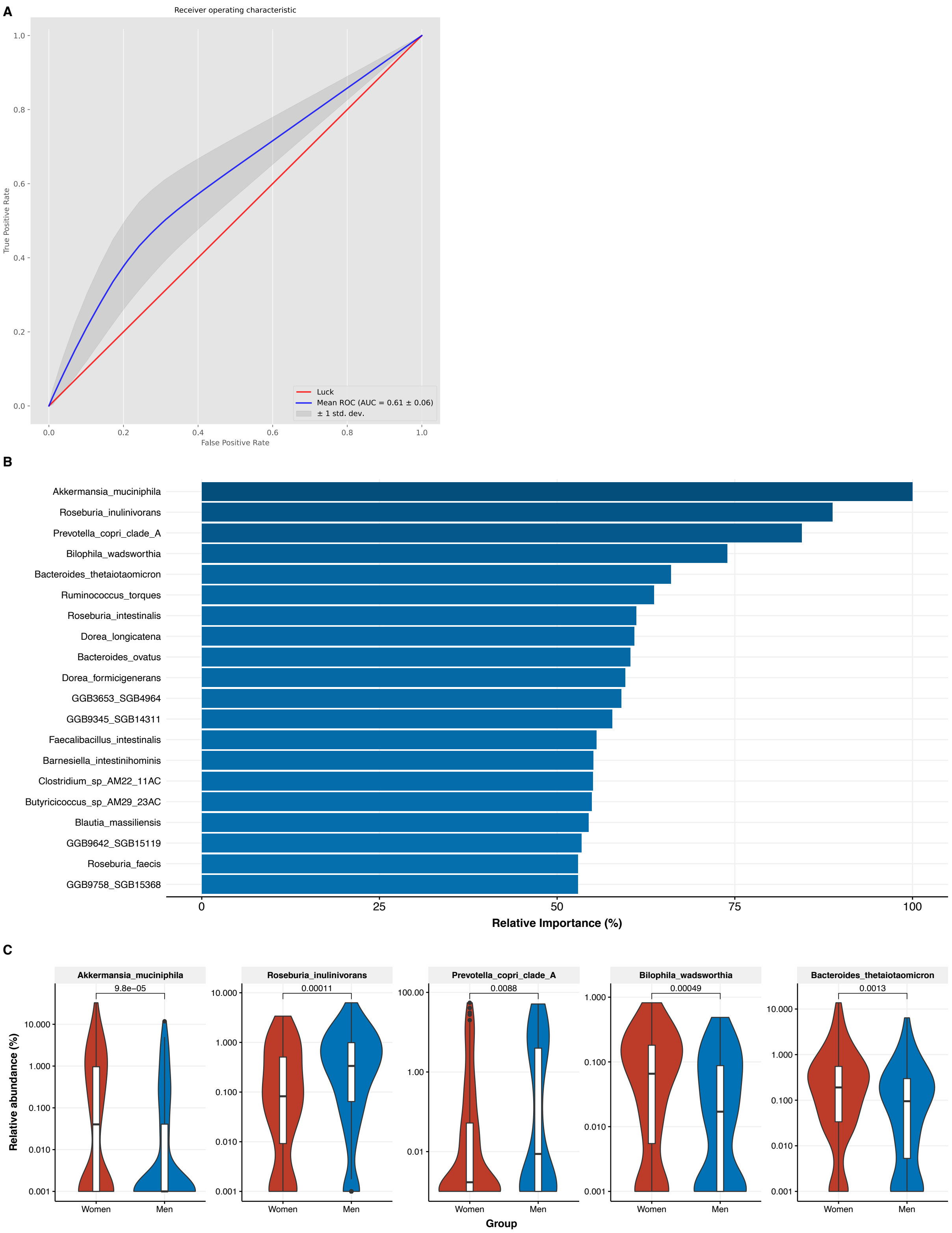

A. Area under the curve (AUC) of the classification model predicting sex from gut microbiota composition derived from metagenomic sequencing data; B. Relative feature importance resulting from the machine learning prediction (first feature always set at 100%, other features shown relative to first); C. Sex differences of highest ranked species (relative abundances + 0.001 plotted on a log-scale) tested with Mann-Whitney U tests.

Supplementary Figure 3: Linear regression models of best predicting microbes for sex using metagenomics data

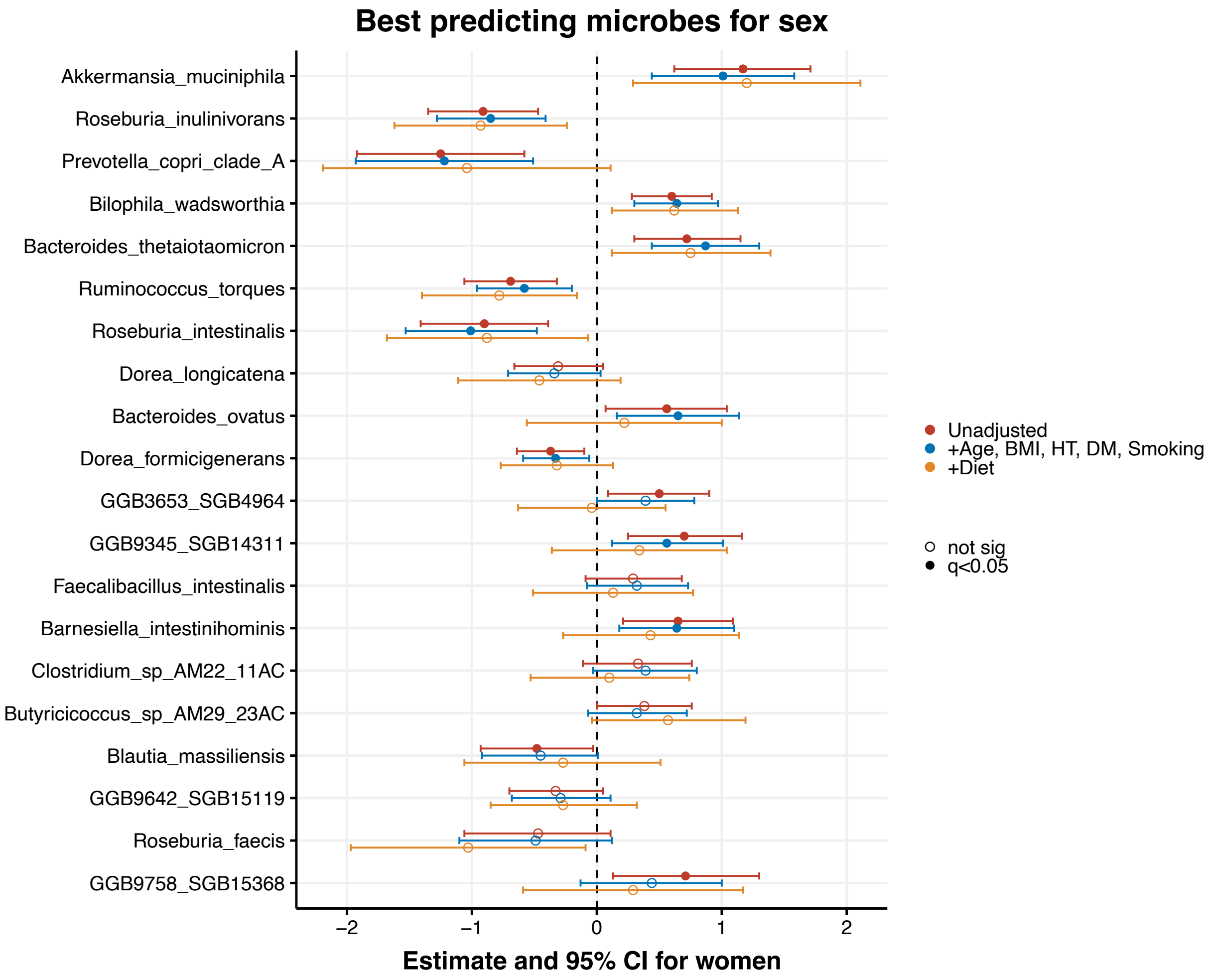

Forest plots of linear regression models with the sex as predictor and log-transformed microbe as outcome using metagenomics data, showing the best predicting microbes for sex, ordered by feature importance in the machine learning model. Estimates with 95% confidence intervals as error bars. Model 1 is unadjusted. Model 2 is adjusted for age, BMI, hypertension (HT), diabetes mellitus (DM) and smoking. Model 3 is additionally adjusted for diet (total calories, alcohol, fibre and protein intake). q-values: p-values adjusted using the Benjamini-Hochberg procedure, q<0.05 was considered significant.

Supplementary Figure 4: Machine learning model: sex differences in functional pathways

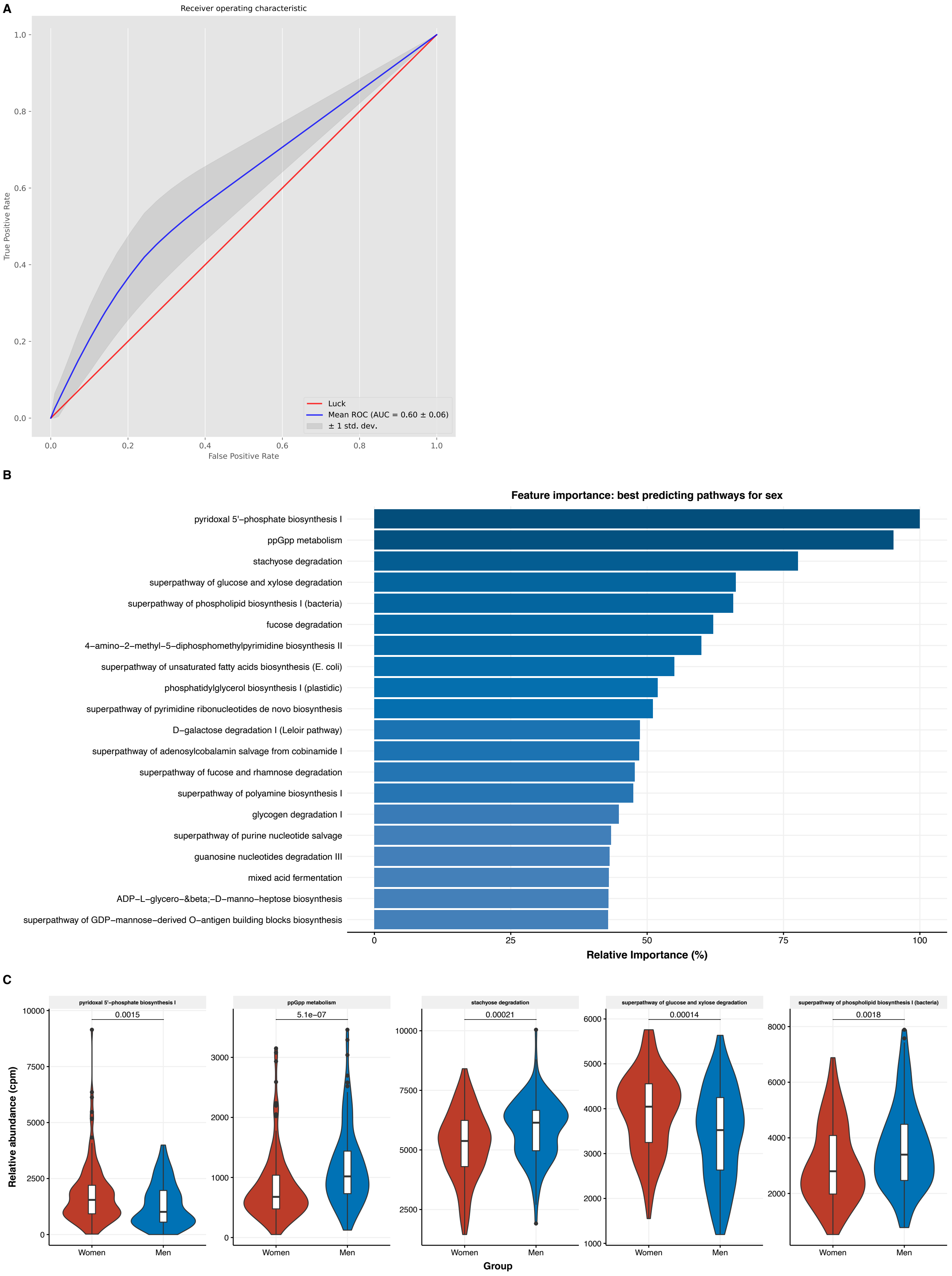

A. Area under the curve (AUC) of the classification model predicting sex from functional pathways derived from metagenomic sequencing data; B. Relative feature importance resulting from the machine learning prediction (first feature always set at 100%, other features shown relative to first); C. Sex differences of highest ranked species in (relative abundances+0.001 plotted on a log-scale) tested with Mann-Whitney U tests.

Supplementary Figure 5: Pyridoxal synthesis pathway stratified per species

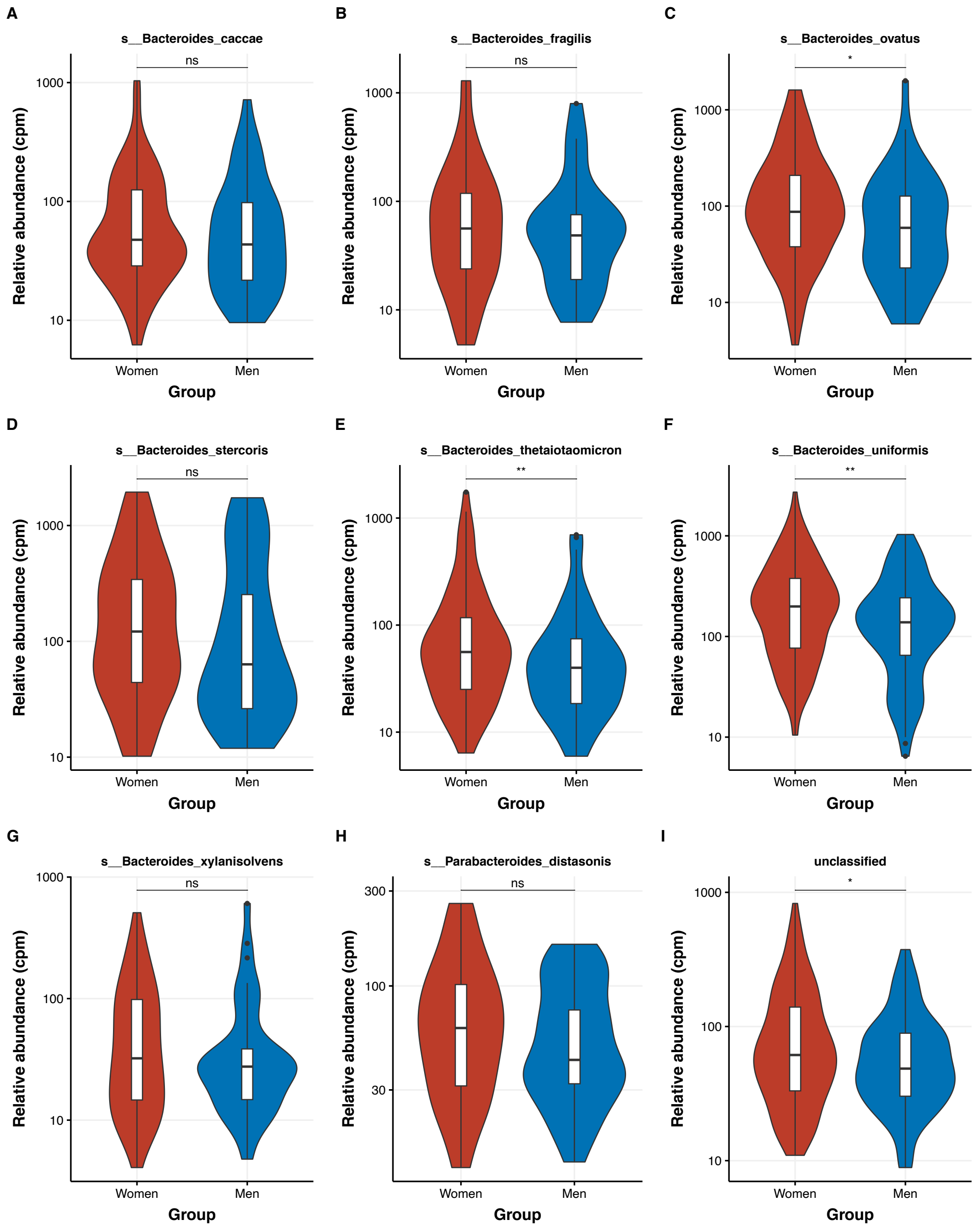

*Sex differences of stratified pathway abundances for the pyridoxal 5'-phosphate biosynthesis pathway per microbial species (relative abundances of pathways in cpm), tested with Mann-Whitney U tests. ns = not significant, * P-value < 0.05, ** P-value < 0.01.*

Supplementary Figure 6: Machine learning model: gut microbiota composition (metagenomics) and menopause

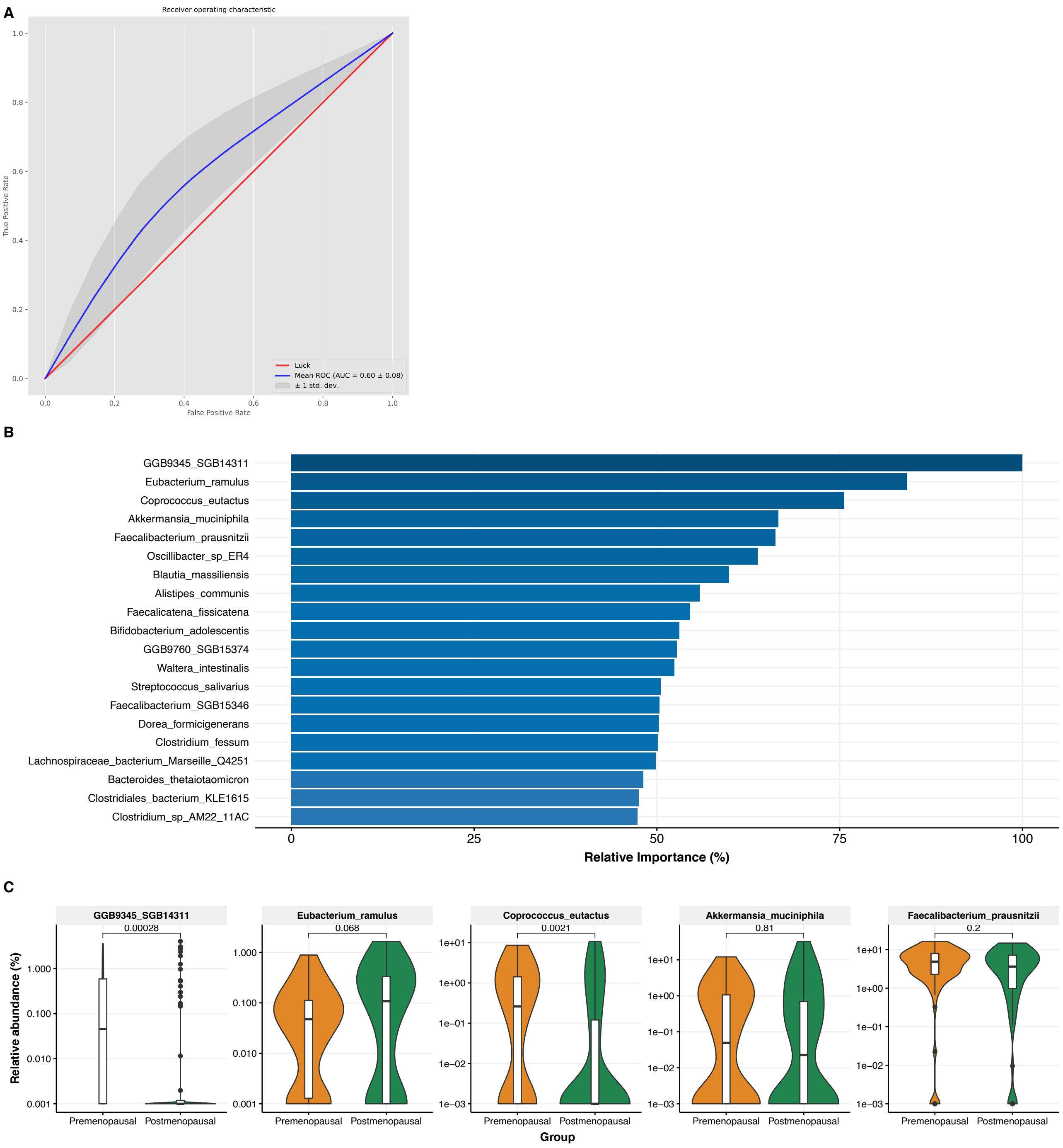

A. Area under the curve (AUC) of the classification model predicting menopause from functional pathways derived from metagenomic sequencing data; B. Relative feature importance resulting from the machine learning prediction (first feature always set at 100%, other features shown relative to

first); C. Menopausal differences of highest ranked species in (relative abundances+0.001 plotted on a log-scale) tested with Mann-Whitney U tests.

Supplementary Figure 7: Linear regression models of best predicting microbes for menopause using metagenomics data

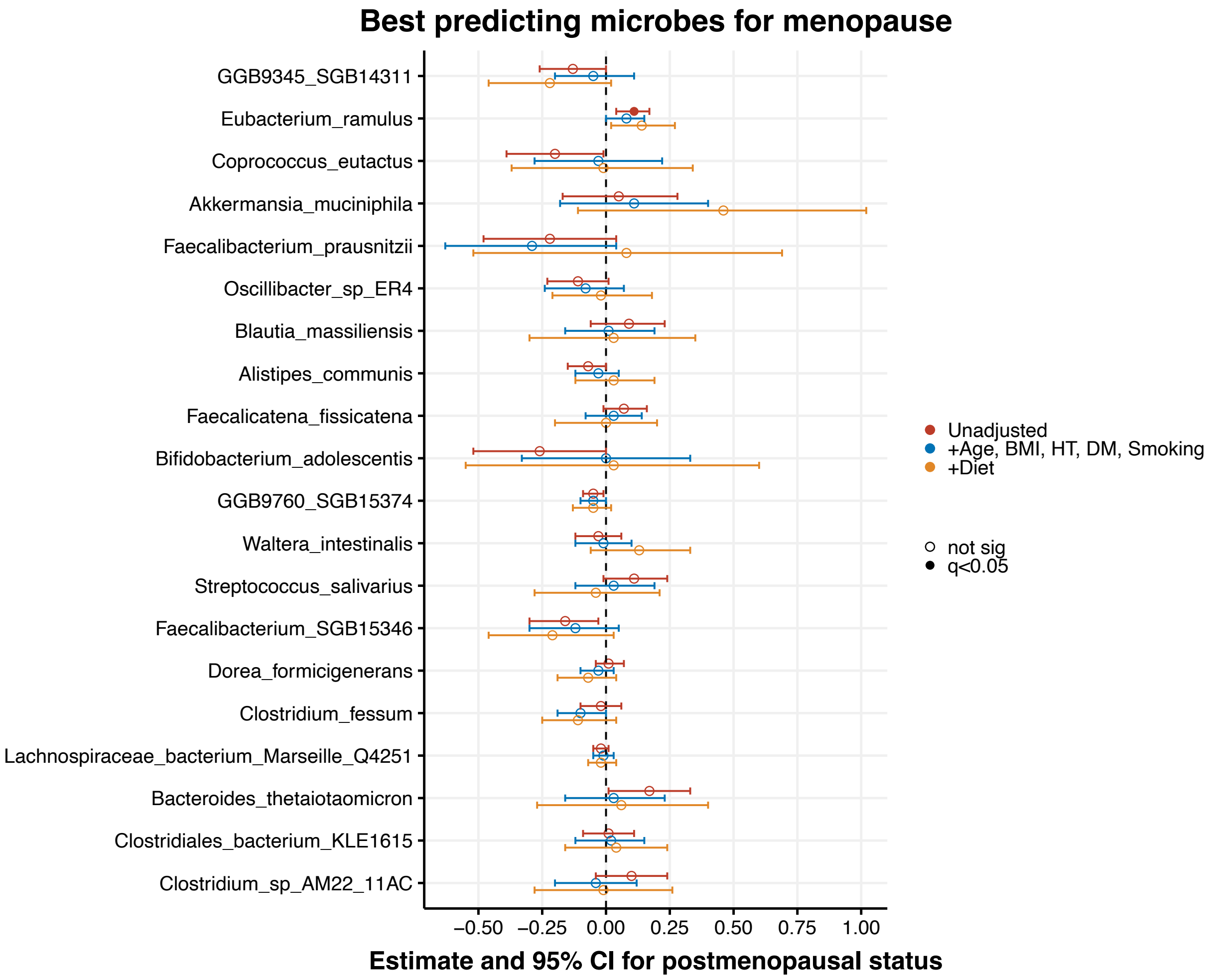

Forest plots of linear regression models with menopausal status as predictor and log-transformed relative abundance of the microbe (+0.01 pseudocount) as outcome using metagenomics data, showing the best predicting microbes for menopausal status, ordered by feature importance in the machine learning model. Estimates with 95% confidence intervals as error bars. Model 1 is unadjusted. Model 2 is adjusted for age, BMI, hypertension (HT), diabetes mellitus (DM) and smoking. Model 3 is additionally adjusted for diet (total calories, alcohol, fibre and protein intake). q-values: p-values adjusted using the Benjamini-Hochberg procedure, q<0.05 was considered significant.

Supplementary Figure 8: Machine learning model: functional pathways and menopause

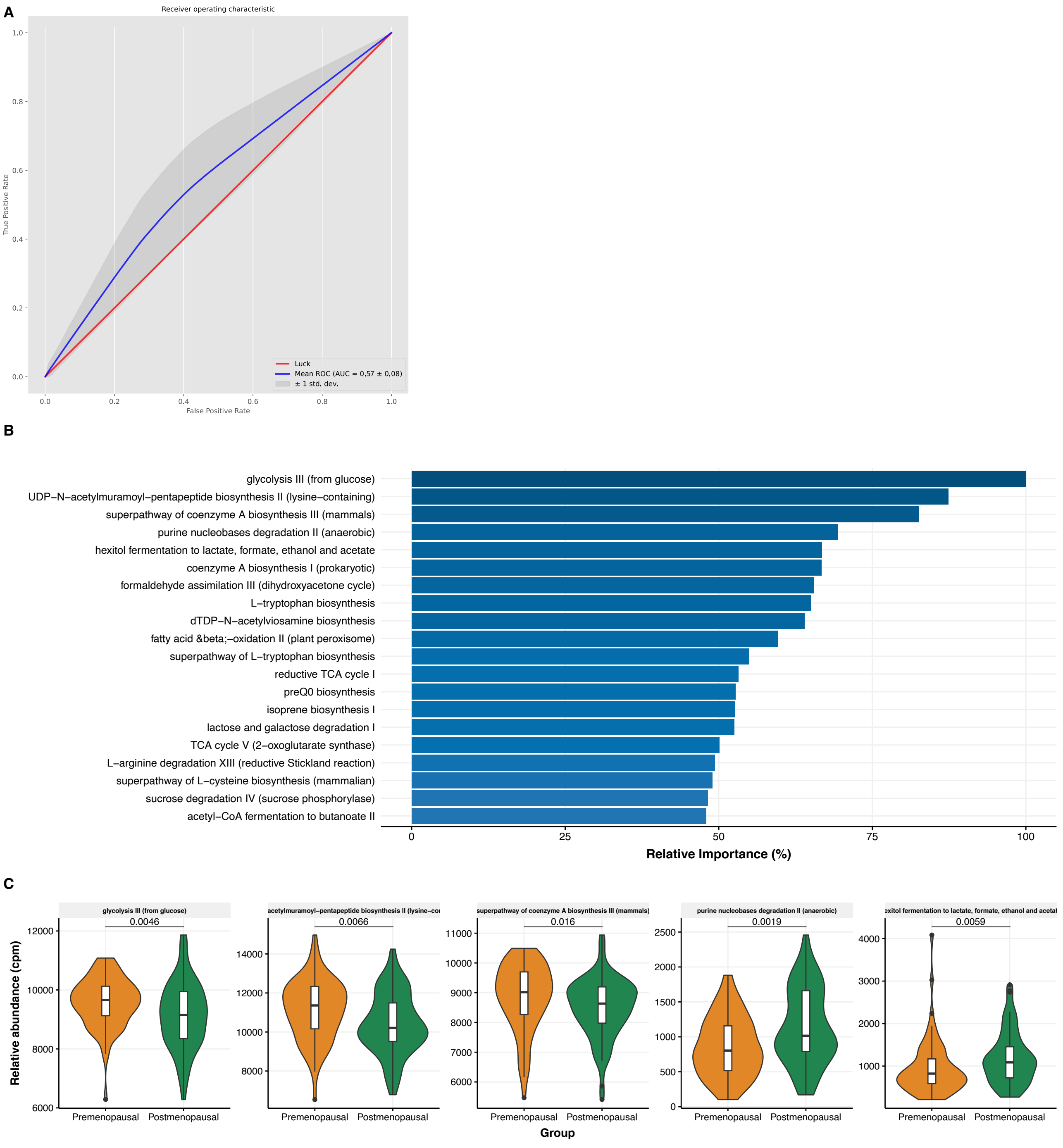

A. Area under the curve (AUC) of the classification model predicting menopause from functional pathways derived from metagenomic sequencing data; B. Relative feature importance resulting from the machine learning prediction (first feature always set at 100%, other features shown relative to first); C. Menopausal differences of highest ranked pathways in abundances in copies per million (cpm) tested with Mann-Whitney U tests.

Supplementary Figure 9: Linear regression models of best predicting pathways for menopause

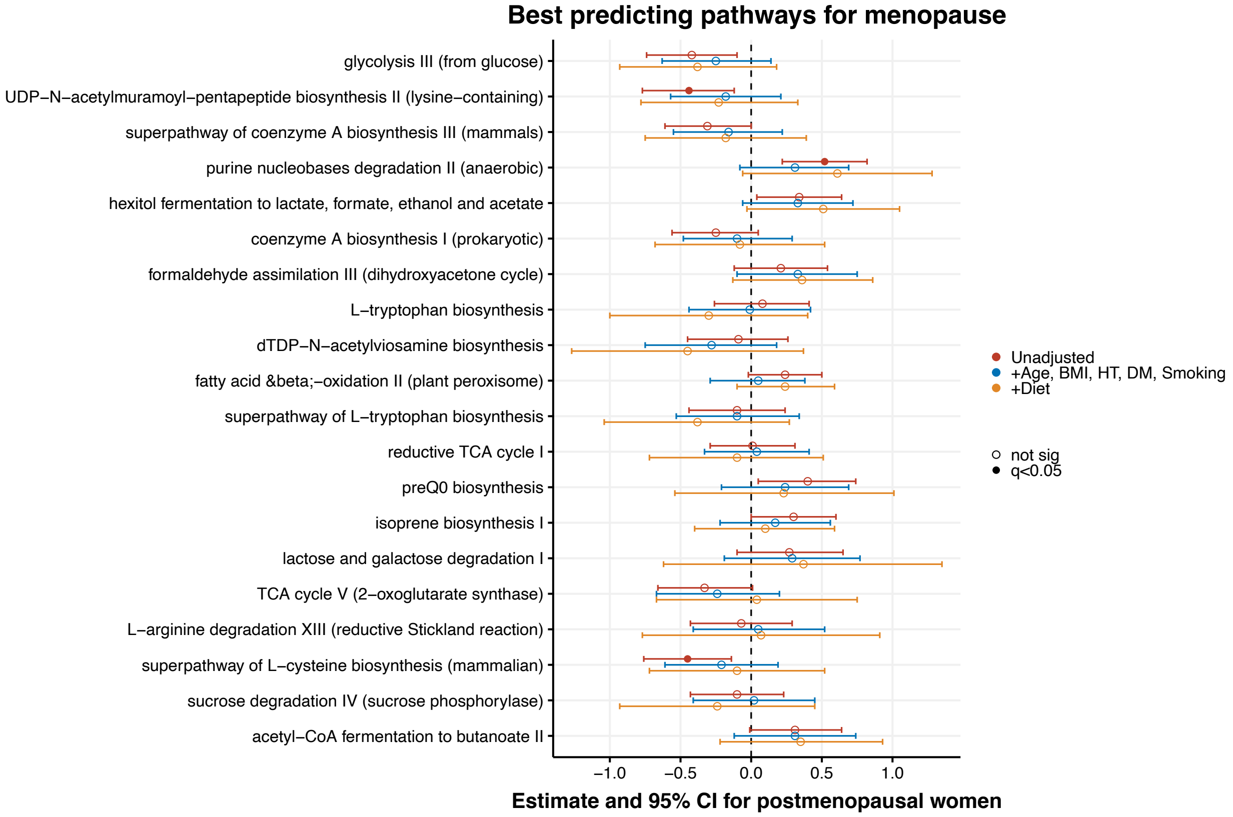

Forest plots of linear regression models with the pathways (abundance scaled to zero-mean-unit-variance) as predictors and menopausal status as outcome, showing the best predicting pathways ordered by feature importance in the machine learning model. Estimates with 95% confidence intervals as error bars. Model 1 is unadjusted. Model 2 is adjusted for age, BMI, hypertension (HT), diabetes mellitus (DM) and smoking. Model 3 is additionally adjusted for diet (total calories, alcohol, fibre and protein intake). BMI = body mass index. HT = hypertension. DM = diabetes mellitus. q-values: p-values adjusted using the Benjamini-Hochberg procedure, q<0.05 was considered significant.

Supplementary Figure 10: Menopausal differences in key pathways

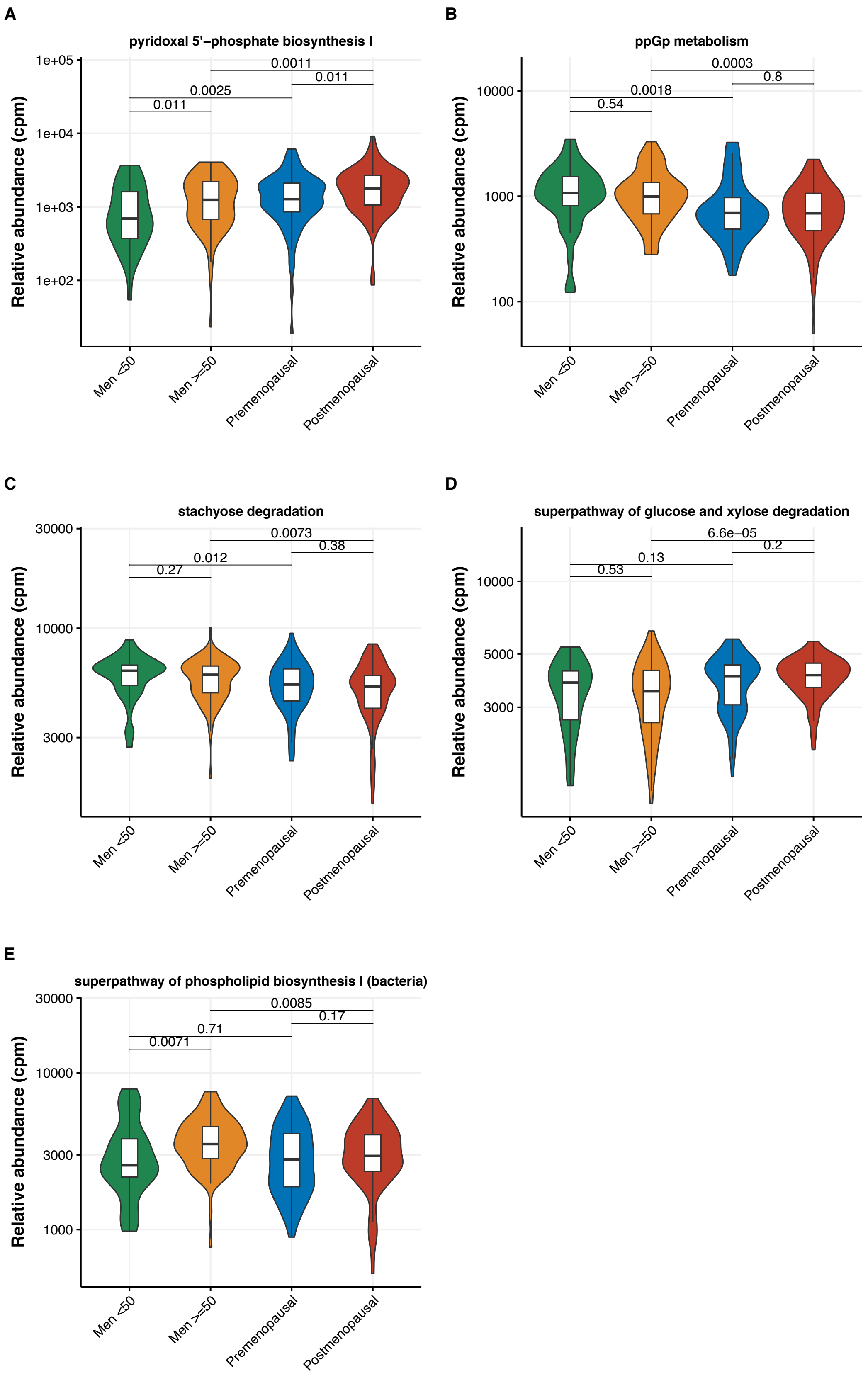

*Comparison of pathway abundances between younger and older men and pre- and postmenopausal women. Differences between groups were tested with Mann-Whitney U tests. * P-value < 0.05, ** P-value < 0.01, *** P-value < 0.001, **** P-value < 0.0001.*
